# Improving arteriovenous fistula patency: Transdermal delivery of diclofenac reduces cannulation-dependent neointimal hyperplasia via AMPK activation

**DOI:** 10.1016/j.vph.2015.02.012

**Published:** 2015-08

**Authors:** Mark G. MacAskill, David G. Watson, Marie-Ann Ewart, Roger Wadsworth, Andrew Jackson, Emma Aitken, Graeme MacKenzie, David Kingsmore, Susan Currie, Paul Coats

**Affiliations:** a Strathclyde Institute of Pharmacy and Biomedical Science, University of Strathclyde Glasgow, UK; b Institute of Cardiovascular & Medical Sciences, University of Glasgow, UK; c Department of Renal Surgery, Western Infirmary, Glasgow, UK

**Keywords:** Arteriovenous fistula Vascular, Re-stenosis, Cell proliferation, Pharmacotherapy

## Abstract

Creation of an autologous arteriovenous fistula (AVF) for vascular access in haemodialysis is the modality of choice. However neointimal hyperplasia and loss of the luminal compartment result in AVF patency rates of ~ 60% at 12 months. The exact cause of neointimal hyperplasia in the AVF is poorly understood. Vascular trauma has long been associated with hyperplasia. With this in mind in our rabbit model of AVF we simulated cannulation autologous to that undertaken in vascular access procedures and observed significant neointimal hyperplasia as a direct consequence of cannulation. The neointimal hyperplasia was completely inhibited by topical transdermal delivery of the non-steroidal anti-inflammatory (NSAID) diclofenac. In addition to the well documented anti-inflammatory properties we have identified novel anti-proliferative mechanisms demonstrating diclofenac increases AMPK-dependent signalling and reduced expression of the cell cycle protein cyclin D1. In summary prophylactic transdermal delivery of diclofenac to the sight of AVF cannulation prevents adverse neointimal hyperplasic remodelling and potentially offers a novel treatment option that may help prolong AVF patency and flow rates.

## Graphical abstract

**Figure:**
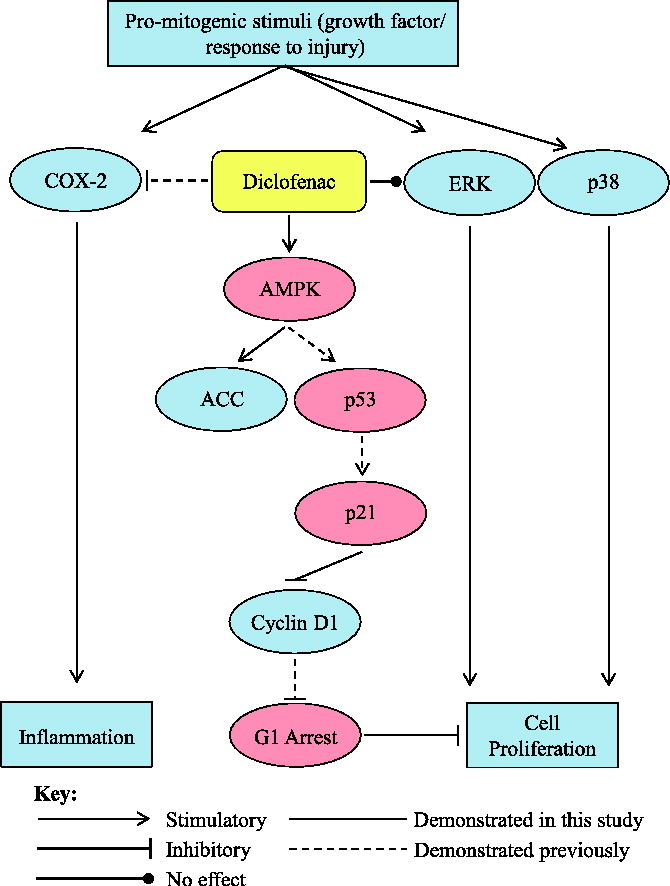

## 1 Introduction

Vascular access is the Achilles heel of modern haemodialysis [1]. The complications of vascular access are responsible for over 20% of all hospitalisations of patients on haemodialysis and account for one third of all in-patient renal bed usage [2]. Autologous arteriovenous fistulae created from native artery and vein are the modality of choice to provide vascular access for haemodialysis. Unfortunately however, native fistulae have poor patency rates. Recently, a systematic review and meta-analysis on AVF patency was published using rigorous methodology to examine 62 unique cohorts [3]. The authors reported that one-quarter to one-third of AVF failed to mature, and by one year 40% of all AVF had failed or required intervention. The hallmark of AVF failure is neointimal hyperplasia leading to stenosis with occlusion of the fistula outflow vein [4,5].

The mechanisms leading to AVF stenosis are not fully understood however interplay between the vascular wall and immune system are important. Cytokines and pro-inflammatory factors have been shown to play central roles in the activation of acute and chronic vascular response to injury [6]. For example enhanced NFκB activation has been shown to result in the expression of a number of pro-inflammatory genes in vitro including iNOS, COX-2, ICAM, VCAM, which have been strongly implicated in neointimal formation [7]. Patients with renal disease undergoing haemodialysis have a raised inflammatory profile with significantly increased hs-CRP, serum TNF-α, IL-1, MCP-1, VCAM-1 and ICAM-1, as well as increased expression of the pro-inflammatory receptor TLR-4 [8–10].

Vascular injury as a consequence of angioplasty or stent insertion is well documented [11]. Central to the haemodialysis procedure is double cannulation of the AVF with a 14/15 G (1.4/1.6 mm diameter) stainless needle 2–3 times every week. The acute trauma inflicted by the needle piercing the vascular wall likely releases numerous pro-inflammatory mediators which promote both wound healing and neointimal growth. There are many studies highlighting the role of pro-inflammatory processes in vein graft failure [12]. The known key signalling pathways driving vascular neointimal proliferation are the mitogen-activated protein kinase (MAPK) pathways; extracellular signal-regulated kinases 1/2 (ERK1/2), c-Jun amino-terminal kinases (JNKs) and the p38 MAPKs [13]. Phosphorylation of ERK is mainly associated with a proliferative response to a mitogenic stimulus, whereas p38 MAPK and JNK pathways are activated by stressors such as hypoxia or injury [14]. Cell proliferation like all active processes within the cell is regulated by a number of upstream co-dependent cellular bioenergetic regulating proteins. A key regulator of cell bioenergetics is AMP-activated protein kinase (AMPK) [15]. Cell energy requirements are significantly raised during cell proliferation and consequently AMPK is activated during AMP: ATP cycling [16].

Currently there are no prophylactic treatments to reduce the progression of neointimal hyperplasia and thrombus formation in AVFs. Percutaneous transluminal angioplasty of stenosis in functioning forearm AVF has been shown to significantly improve patency and decrease access-related morbidity [17]. However, the disadvantages of these procedures are that they require frequent revision as the 12 month patency rate can be as low as 26% [18]. The option of pharmacotherapy to reduce AVF stenosis in this patient group is also challenging. Co-morbidities, impaired hepatic metabolism, profoundly impaired or no renal excretion and adverse bleeding events limit this as a clinically viable option [19].

The considered use of non-steroidal anti-inflammatory drugs (NSAIDs) which have anti-proliferative and anti-inflammatory activity may offer a potential option as an interventional treatment for AVF neointimal hyperplasia and thrombus formation. Thus the aim of this study was to investigate for the first time the efficacy of transdermal delivery of the NSAID diclofenac in reducing neointimal hyperplasia and thrombus formation as a consequence of cannulation-dependent injury of the AVF.

## 2 Materials and methods

### 2.1 Creation of the femoral arteriovenous fistula

Ethical approval for the use of animals in this study was obtained from University of Strathclyde Ethics Committee. The licence to undertake in-vivo work was granted by the UK Home Office in accordance with the Animals (Scientific Procedures) Act 1986.

Rabbits (New Zealand White) were given a pre-medication of hypnorm (0.3 ml/kg, VetaPharma Ltd., UK) intramuscularly 15 min prior to surgery. During surgery the rabbit's body temperature, respiration and heart beat were visually observed and recorded. Based on respiration and heart rate, the concentration of isoflurane was adjusted between 1 and 1.5%. Subcutaneous Rimadyl (4 mg/kg, Pfizer, UK) was also given at the time of surgery. A site proximal to the right knee was chosen to create the AVF due to the superficial nature of the vessels. The subsartorial femoral artery and vein were mobilised and flushed with IV heparin 1000 units given via the marginal ear vein. The vessels were controlled with (8 mm) clamps and 5 mm longitudinal arteriotomy and venotomy performed. A side-to-side anastomosis using 10–0 polyamide suture (Ethicon, USA) was created. The distal vein was then ligated using a 4–0 polyamide suture (Ethicon, USA) to ensure unidirectional blood flow through the vein, in effect creating an end to side anastomosis.

### 2.2 Ultrasonography of arteriovenous fistula

Ultrasound measurements were performed using a Philips Sono 5500 and a linear array probe (6–15 mHz) 10 days following arteriovenous creation. Brightness mode (B-mode) ultrasound was used to visualise the patency of vessels and to measure vessel diameter. Pulsed Wave Doppler mode was used to measure the blood velocity. Blood flow (ml/min) was calculated.

### 2.3 Cannulation injury and diclofenac application

Cannulation injury to the venous branch of the AVF was performed three times a week during days 28–56 by the same individual. Needle stick injury was confined to an area of 1–1.5 cm along the outflow vein, mimicking the area cannulation technique used clinically. A saline charged 23 gauge needle was inserted into the vein for 60 s after which the needle was withdrawn and pressure placed on the AVF. At the time of cannulation, ultrasound was used to ensure placement of the needle within the vein. No flow was applied through the cannula. Diclofenac diethylammonium gel (1.16%, Novartis, UK) was applied topically to the AVF cannulation site in treatment group animals days 28–56. Animals received 750 mg (equivalent to 1 mg/cm^2^ dose of diclofenac) twice a day.

### 2.4 Measurement of serum diclofenac

Analysis of the samples was carried out by injecting 10 μl onto an ACE C18 column (3 mm × 150 mm, 3 μm, HiChrom, Reading UK) with a mobile phase consisting of 1 mM acetic acid in water/1 mM acetic acid in acetonitrile (30:70) at a flow rate of 0.4 ml/min. The run time was 7 min. Detection was carried out by using an Orbitrap Exactive instrument in negative ion mode with a needle voltage of − 4 kV and sheath gas and auxiliary gases set at 50 and 17 arbitrary units respectively. The instrument was scanned between 100 and 700 amu and tuned so that the accurate masses of the analytes were within 3 ppm of the exact masses.

Calibration curves were constructed by spiking 20 ng of flurbiprofen (IS) into 6 × 0.5 ml aliquots of serum and a series consisting of 0, 1, 2, 4, 8, 16 ng of diclofenac. The protein was then precipitated by adding 0.5 ml of acetonitrile, centrifuging and removing the supernatant for analysis. Aliquots of the biological samples (0.2 ml) were treated in the same way but were spiked with 8 ng of IS and protein was precipitated with 0.2 ml of acetonitrile. The 0 point showed no interfering peak for diclofenac and the 1 ng/0.5 ml spike showed a clear peak with a peak height of around 50,000. The cut off point for the trap is 5000 so the limit of detection was 0.1 ng/0.5 ml.

### 2.5 Histological processing of rabbit AVF

Following euthanasia by intravenous injection of sodium pentobarbital (1 ml/kg), the animal's vessels were perfusion fixed with 4% paraformaldehyde. The AVF and contralateral control vessels were removed with surrounding muscle still attached and placed in fresh 4% paraformaldehyde solution. Tissues were then wax processed and cut at 5 μM before being stained with haematoxylin and eosin.

### 2.6 Vascular smooth muscle cell culture

Femoral vein was isolated from white New Zealand rabbits. Mouse wild type and AMPKα1^−^/^−^ aortas were isolated from 8 week old sv129 mice. Vessels were cut into rings and VSM cell explants cultured in 1:1 waymouths: ham's F12 (Gibco, UK) with 15% foetal calf serum (FCS) (Biosera, France), 1% penicillin streptomycin (Gibco, UK at 37 °C, 5% CO_2_, 100% humidity).

### 2.7 ^3^H-thymidine incorporation assay

Cell proliferation was determined using serum-induced [^^3^^H]-thymidine incorporation as described previously [20]. Quiesced cells were stimulated with 10% FCS for 24 h, with the addition of 1 μCi/well of ^3^H thymidine (Amersham, UK) for the final 6 h. Cells were washed with ice cold PBS and four washes of 10% trichloroacetic acid. Thereafter cells were solubilised with 250 μl lauryl sulphate (10%) plus sodium hydroxide (0.2 M). The contents of each well were then transferred to a vial and radioactivity quantified by liquid scintillation in DPM-1 using a Packard 1500 Tri-Carb (PerkinElmer, USA).

### 2.8 Immunoblotting of VSM cell lysates

Treated/stimulated VSM cells were washed with ice cold PBS and lysed by addition of boiling sample buffer (0.125 M Tris–HCl (pH 6.7), 0.5 mM Na_4_P_2_O_7_, 1.25 mM EDTA, 1.25% (v/v) glycerol, 0.5% (w/v) SDS, 25 mM dithiothreitol and 0.02% (w/v) bromophenol blue). Samples were loaded on to a 10% SDS page gel (minimum of 20 μg total protein per well), and following electrophoresis proteins were transferred to a nitrocellulose membrane. Membranes were blocked for 1 h at room temperature in 3% BSA and incubated overnight in the following primary antibodies (1:1000); anti-phospho/Total ERK rabbit IgG (Cell Signalling Technology, USA), anti-phospho/Total p38 rabbit IgG (Cell Signalling Technology, USA), anti-Cyclin D1 rabbit IgG (Santa Cruz Biotechnology Inc., USA), anti-GAPDH mouse IgG (Cell Signalling Technology, USA), anti-phospho/Total AMPK rabbit IgG (Cell Signalling Technology, USA), and anti-phospho/Total ACC rabbit IgG (Cell Signalling Technology, USA). For detection of the primary antibody, a HRP-conjugated anti-rabbit IgG at 1/2000 dilution (Cell Signalling Technology, USA) or anti-mouse IgG at 1:10,000 dilution (Stratech Scientific Ltd, UK) was used. The blots were developed using an enhanced chemiluminescence kit (Thermo Scientific Peirce, USA).

### 2.9 Statistical analysis

All in vitro results are shown as the mean relative to control. Minitab was used to carry out statistical analysis, with p < 0.05 considered significant. A general linear ANOVA model with cells from each separate animal entered as a random factor was used to assess the effect of increasing diclofenac concentrations; and post hoc Dunnett's used to assess the significance of specific treatments vs. control. Paired and unpaired T-tests were used for single comparisons, and a one way ANOVA used for comparison of multiple groups within in-vivo studies.

## 3 Results

### 3.1 Conformation of AVF function and maturation

**Fig. 1:**
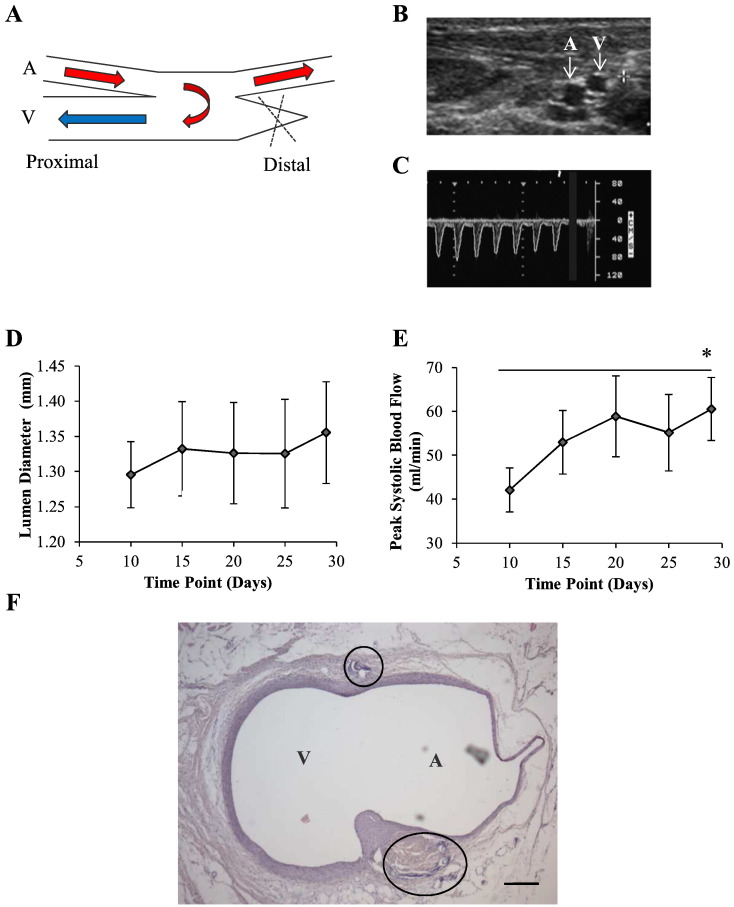
Creation and maturation of rabbit femoral AVF. A, AVF between the femoral artery and vein. The anastomosis was created by continuous suture (10–0) in a side to side fashion, followed by ligation of the distal vein. B, B-mode ultrasound was used to visualise patency of the AVF following creation. C, Pulsed Wave Doppler was used to measure the velocity of blood within the AVF. A pulsatile waveform was present in the vein, indicating successful flow of arterial blood through the AVF. D, Over 4 weeks post-AVF creation, venous lumen diameter and E, peak blood flow significantly increased. Results are shown as the mean ± S.E.M., n = 11, * = p < 0.05 (paired T-test for day 10 vs. day 30). F, Histological examination (H&E) of the anastomosis 8 weeks following AVF creation reveals healthy integration of both vessels with intact vascular wall. Scale bar = 200 μm, A = artery, V = vein.

Following the creation of an AVF between the femoral artery and vein, blood flow was restored to allow arterial blood to enter directly into the femoral vein as shown in Fig. 1A. Patency and lumen diameter of both vessels was monitored by cross sectional B-mode ultrasound (Fig. 1B). Over the first 4 weeks post-AVF creation, venous lumen diameter showed a small increase (Fig. 1D). The velocity of blood within the vein, measured by Pulsed Wave Doppler, revealed a pulsatile waveform originating from the artery (Fig. 1C). Using these techniques, blood flow through the vein was calculated (Fig. 1E). At the end of the maturation period there was a significant time dependent rise in blood flow, increasing from 42.1 ± 5.0 ml/min at days 10 to 60.5 ± 7.2 ml/min at day 28. At the end of each study, vessels were perfusion fixed in situ and stained using H&E. Fig. 1F shows an example of the anastomosis site revealing healthy integration of the artery and vein with intact vascular wall with no focal remodelling or thrombosis.

### 3.2 Effect of needle cannulation and diclofenac treatment on AVF structure and function

**Fig. 2:**
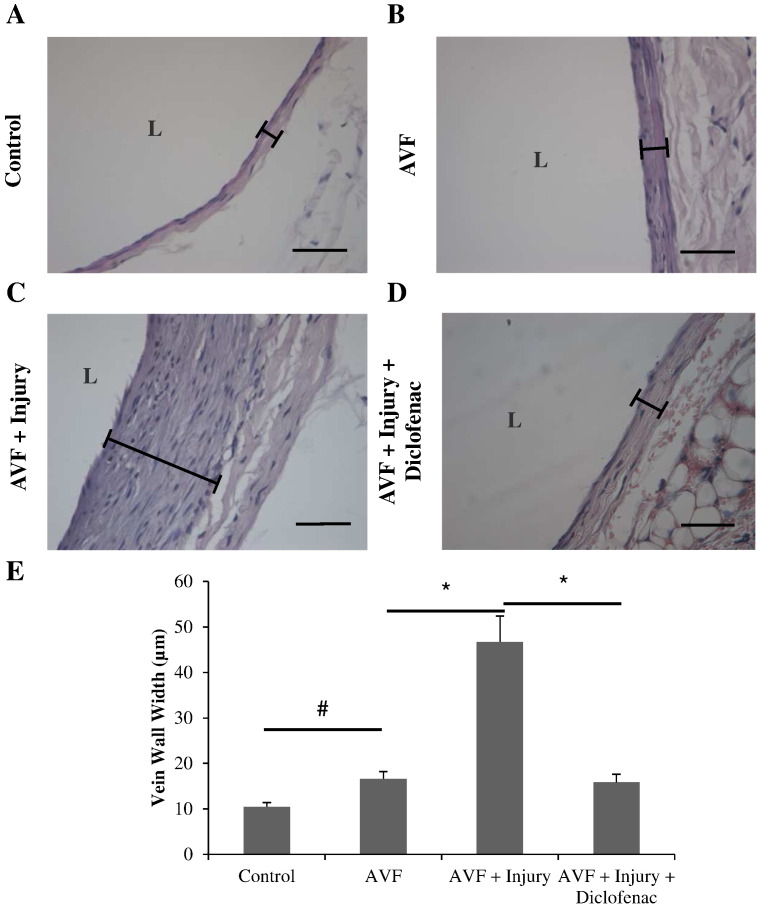
Shows venous wall remodelling following creation of AVF A, control un-operated femoral vein, B, AVF, C, AVF with cannulation injury and D, injured AVF with diclofenac treatment (scale bar A–D = 50 μm, L = lumen). E, Vascular remodelling was quantified by measuring vein wall thickness for each group (defined in examples by bar). Creation of an AVF was associated with a small but significant increase in remodelling. Injury resulted in a significantly higher degree of remodelling which was inhibited by topical application of diclofenac. Results are shown as the mean ± S.E.M. n = 7, 7, 6 & 6 for control, AVF, AVF + Injury and AVF + Injury + Diclofenac respectively. # = p < 0.05 unpaired T-test for unoperated control vs. AVF, * = p < 0.05 one way ANOVA with post hoc Bonferroni's correction for AVF vs. AVF + Injury & AVF + Injury vs. AVF + Injury + Diclofenac.

In the second phase of the study, animals were split into a control, non-cannulated group, a group that underwent cannulation and a group that underwent cannulation and diclofenac treatment. The effect of AVF creation, cannulation and diclofenac intervention on vascular remodelling within the venous branch of the AVF was assessed histologically (Fig. 2). The creation of an AVF resulted in a significant increase in mean vein wall width from 10.5 ± 0.9 μm to 16.6 ± 1.6 μm (Fig. 2B & E). In the cannulation group there was a significant 2.8 fold increase in vein wall thickness vs. non-cannulated control (Fig. 2C & E). This remodelling consisted of an increased cellular component as well as degradation of the vein wall at the adventitial side of the media. In the cannulation plus diclofenac treatment group there was a significant three fold decrease in vein wall thickness when compared to cannulation alone (Fig. 2D & E). Serum concentration of diclofenac measured on days 29 and 56 were 1.6 ± 1.4 and 74.1 ± 14.8 ng/l respectively.

### 3.3 Effect of diclofenac on rabbit VSM cell proliferative mechanisms

**Fig. 3:**
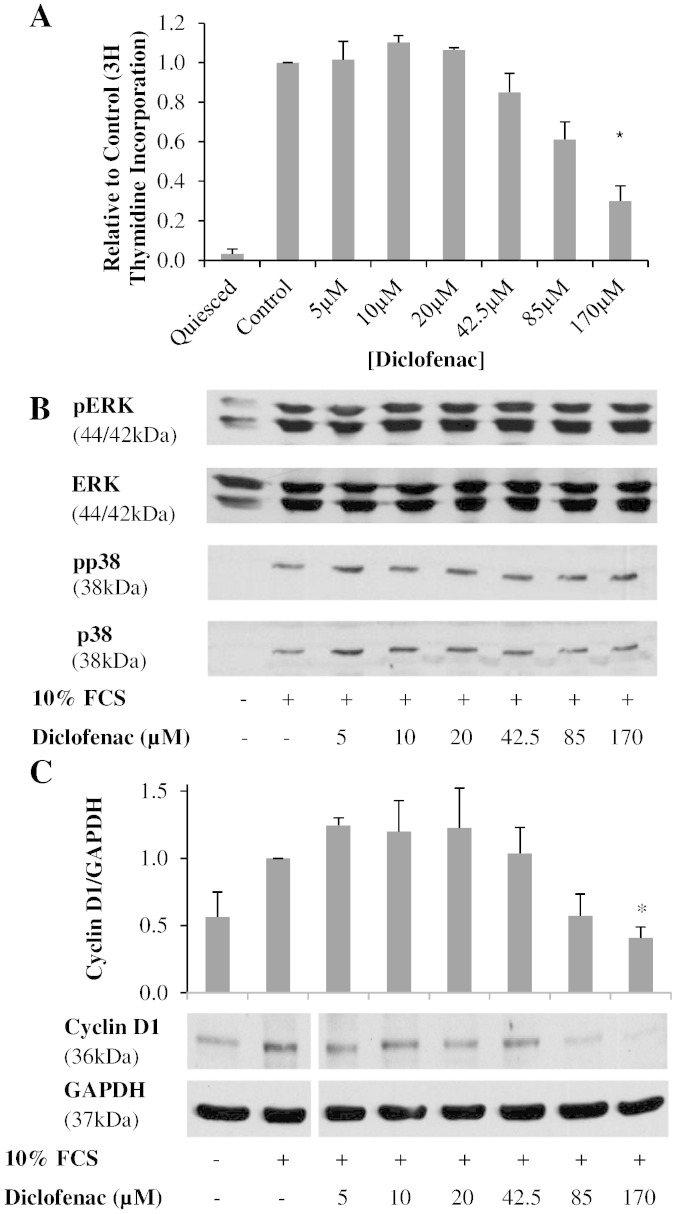
Shows the effect of diclofenac on key cell signalling events associated with cell proliferation. A, Quiesced VSM cells were treated with diclofenac and stimulated for 24 h with 10% FCS. Proliferation, measured by ^3^H thymidine incorporation, was reduced by diclofenac treatment in a concentration dependent manner (general linear ANOVA, p < 0.05). Results are shown as the mean relative to control ± S.E.M., n = 4, * = p < 0.05 (post-hoc Dunnett's vs. control). B, Diclofencac treated VSM cells were stimulated with 10% FCS for 15 min and immunoblotting was carried out for ERK or p38 phosphorylation. Diclofenac had no effect on the activation of these MAPK. C, Following diclofenac treatment and stimulation with 10% FCS for 8 h, cyclin D1 expression was reduced in a concentration dependent manner. Results are shown as the mean ± S.E.M., n = 6, general linear ANOVA p < 0.05, * = post-hoc Dunnett's vs. control p < 0.05.

The anti-proliferative activity of diclofenac on rabbit VSM cells was analysed by ^3^H-thymidine incorporation. Based on reported anti-proliferative concentrations diclofenac pre-treatment [5 μM–170 μM] caused a concentration dependent decrease in ^3^H-thymidine incorporation as shown in Fig. 3A [21]. Further to this the effect of diclofenac on MAPK kinase activation was investigated by immunoblot (Fig. 3B). Under basal conditions phosphorylation of ERK was high, although the cells still had the capacity for further phosphorylation when stimulated by 10% FCS. Diclofenac pre-treatment had no effect on FCS stimulated ERK activation. Under basal conditions p38 was not expressed. However upon stimulation with 10% FCS both total and phosphorylated p38 increased which, like the ERK response, was not affected by diclofenac. As it has been previously demonstrated that diclofenac causes an accumulation in G1 of the cell cycle, cyclin D1 expression was also investigated (Fig. 3C) [21]. Diclofenac caused a significant dose dependent decrease in cyclin D1 expression leading to a 60% reduction at the highest concentration (170 μM).

### 3.4 Effect of diclofenac on AMPK signalling

**Fig. 4:**
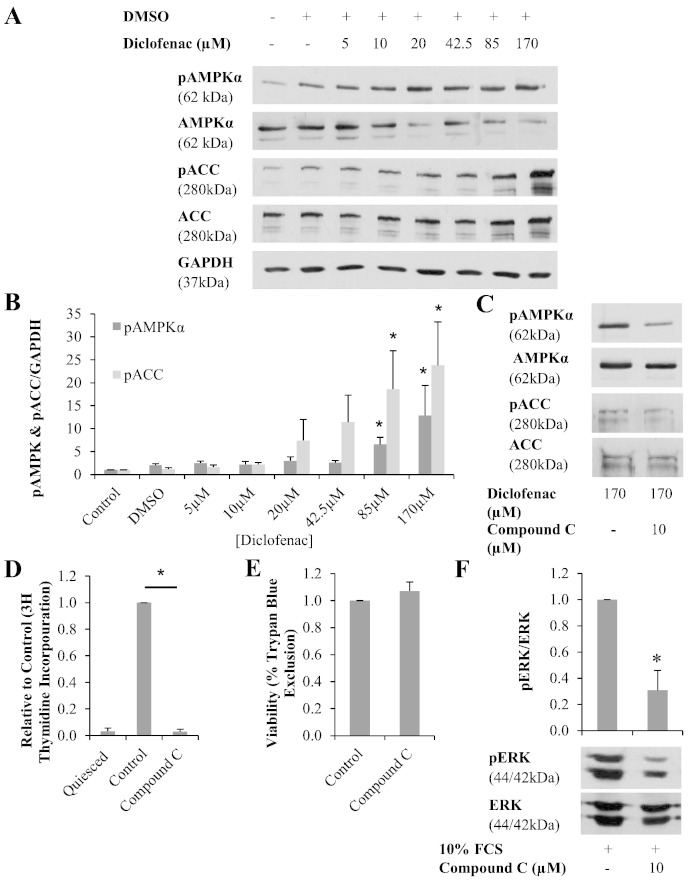
Shows the effect of diclofenac on the AMPK-dependent signalling pathway. A, Following a 24 hour incubation with diclofenac, phosphorylated AMPK, and downstream ACC, increased in a concentration dependent manner. B, Densitometry of AMPK blots, results are shows as the mean relative to untreated control, ± S.E.M., n = 6, * = p < 0.05 (general linear ANOVA with post-hoc Dunnett's vs. control). C, AMPK antagonist compound C inhibits diclofenac mediated activation of AMPK and ACC. D, In contrast to what was expected compound C reduces cell proliferation (n = 4, * = p < 0.05 paired T-test), E, without effecting cell viability (n = 4) F, via AMPK independent inhibition of ERK (n = 3, * = p < 0.05 paired T-test).

The effect of diclofenac on AMPK phosphorylation, and activation of downstream ACC was assessed. Phosphorylation of AMPK was detected under basal conditions (Fig. 4A) and increased significantly following diclofenac treatment in a dose dependent manner. At 175 μM diclofenac, AMPK phosphorylation increased by 13 fold when compared with untreated controls (Fig. 4B). In addition, increased phosphorylation of the AMPK substrate ACC was evident (Fig. 4A). Phosphorylation of ACCα and ACCβ isoforms were detected and total pACC was increased by 24 fold vs. untreated controls following treatment with 175 μM diclofenac (Fig. 4B).

### 3.5 The role of AMPK in the modulation of proliferation by diclofenac

To confirm the role of AMPK in the anti-proliferative activity of diclofenac, the pharmacological AMPK inhibitor compound C was assessed. Treatment with compound C (10 μM) significantly reduced AMPK phosphorylation by 49% (Fig. 4C). Downstream of this, diclofenac mediated phosphorylation of ACC by AMPK was also reduced by 60% (Fig. 4C). Following confirmation of AMPK inhibition by compound C, the effect of this inhibitor on the activity of diclofenac was assessed using ^3^H thymidine incorporation. Surprisingly, 10 μM compound C alone caused cell proliferation to reduce to baseline levels (Fig. 4D) without affecting cell viability as assessed by trypan blue exclusion (Fig. 4E). On further investigation, compound C was shown to inhibit ERK phosphorylation, indicating an effect non-specific to AMPK (Fig. 4F).

**Fig. 5:**
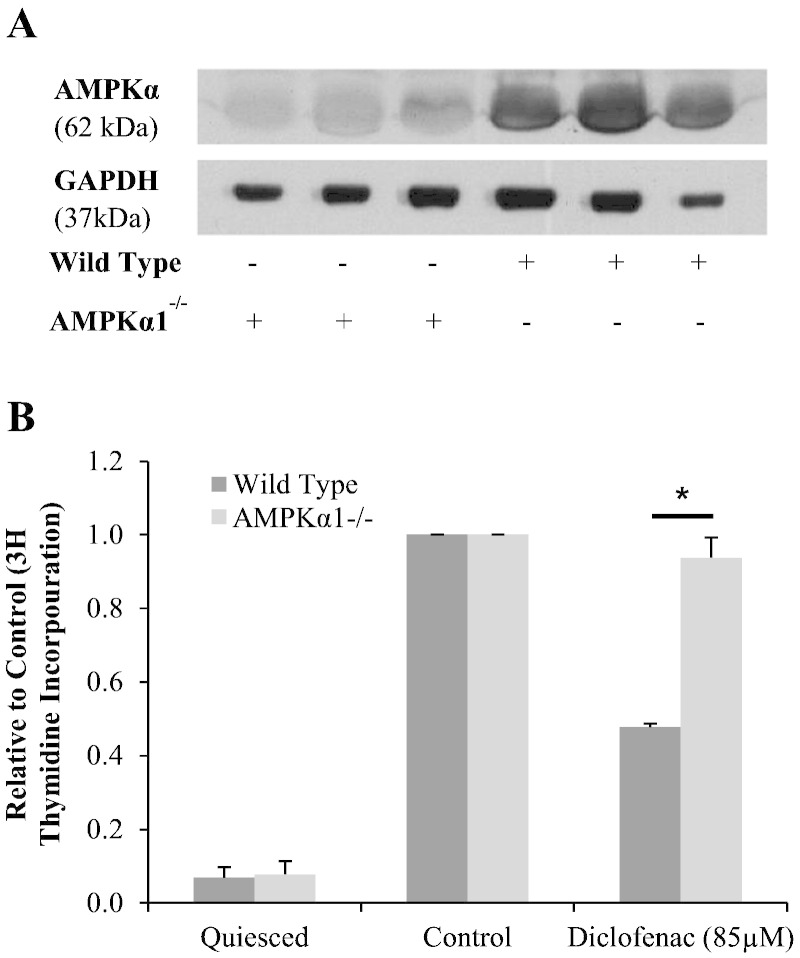
Highlights the AMPKα1-dependent action of diclofenac. A, VSM cells were explanted form wild type and AMPKα1^−^/^−^ sv129 mouse aorta. AMPKα1 knock out was confirmed by immunoblotting. B, Mouse VSM cells were treated with 85 μM diclofenac for 24 h before being stimulated with 10% FCS. AMPKα1^−^/^−^ VSM cell proliferation was not affected by diclofenac treatment, unlike wild type proliferation which was reduced by half. Proliferation was measured by ^3^H thymidine incorporation and expressed as the mean relative to control, ± S.E.M., n = 4, * = p < 0.05 (unpaired T-test).

As compound C exhibited AMPK-independent activity, an alternative approach to inhibit AMPK by siRNA repression was attempted. However, consistent rundown of AMPK could not be achieved and this method proved unsuitable (data not presented). In order to circumvent this issue, VSM cells were explanted and cultured from sv129 wild type and AMPKα1^−^/^−^ mouse aortas. In addition to mouse genotyping, expression of AMPKα was carried out (Fig. 5A). As expected, cells explanted from AMPKα1^−^/^−^ aortas had significantly reduced levels of AMPKα expression. Having confirmed this reduction in AMPK, the effect of diclofenac treatment on ^3^H thymidine incorporation in of wild type vs. AMPKα1^−^/^−^ cells was assessed (Fig. 5B). Diclofenac (85 μM) inhibited wild type cell proliferation by 52%. In contrast, cell proliferation was only inhibited by 6% inhibition in AMPKα1^−^/^−^ cell (p = 0.001). Interestingly, when AMPKα1^−^/^−^ cells were treated with 170 μM diclofenac, cell viability was dramatically reduced. This was, not the case in the wild type cells (data not presented).

## 4 Discussion

In this study we have investigated the effect of repeated needle-dependent injury on AVF structure and function. The function of the AVF for vascular access in haemodialysis is dependent on high blood flow and thus a patent lumen is vital to this procedure. In this study we have demonstrated for the first time that the procedural practice of cannulation, as used in gaining vascular access in haemodialysis, drives a vascular proliferative response resulting in luminal occlusion, reduced blood flow and ultimately failure in terms of vascular access and haemodialysis (Fig. 2).

In our rabbit model as expected the vein section of the AVF underwent an adaptive remodelling response following creation of the fistula and the introduction of arterial blood pressure/flow. Cannulation injury in a frequency similar to that which is undergone in dialysis caused a significant increase in wall thickness at the cannulation site. In the uninjured AVFs no such remodelling was observed thus indicating cannulation injury may be central to the adverse remodelling and focal stenosis observed clinically in AVFs. Re-stenosis in response to vascular injury including angioplasty, stent insertion and surgery is well established and there are a number of established pharmaco-therapeutic interventions to counteract restenosis [21,22]. However what is noteworthy about restenosis and occlusion in patients with renal failure and AVFs is the rapid time to occlusion when compared with lower limb or coronary restenosis events [3]. When considered, this patient cohort on renal dialysis with a documented raised inflammatory profile as a consequence of uraemia related factors and other co-morbidities combined with the frequent and sustained needle insertion may go some way to explain the rapid progression to AVF occlusion [23,24].

To date there is no prophylactic intervention to offset the adverse remodelling associated with needle cannulation injury. In terms of pharmacotherapy we considered that this patient group would have greatly reduced renal function. We therefore opted for transdermal delivery at the site of needle cannulation in our experimental animal model. NSAIDs have previously been demonstrated to be of worth in this patient cohort in terms of increasing AVF patency. However, there exists no convincing evidence to suggest that oral NSAIDs, such as aspirin, alter the underlying mechanism of intimal hyperplasia, but instead have a minor benefit by inhibiting thrombosis. In this present study, transdermal delivery of diclofenac resulted in a significant reduction of needle-dependent-AVF remodelling when compared with untreated AVFs (Fig. 2). This, to the best of our knowledge is the first time that such a potential treatment intervention has been demonstrated. The transdermal route of delivery offers a number of advantages; site directed, permeates the vascular wall, reduces toxicity/adverse bleeding and importantly the patient where appropriate can self-administer.

The approved maximum daily dose for Voltaren Gel 1% is 32 g (1280 mg diclofenac equivalent) and is less than the highest daily dose tested by Kienzler et al (2010) who compared diclofenac bioavailability of oral vs. topical routes of administration [25]. In this present study 2 g (80 mg equivalent diclofenac) applied topically to the cannulation site had profound effect on the adverse remodelling. However, with complete inhibition of cannulation-dependent remodelling with the dose delivered, albeit relatively low, this could potentially be further reduced in an attempt to find a true minimum effect dose. The prescribing of NSAIDs for subjects undergoing dialysis is greatly discouraged [26]. Preservation of any residual renal function, as well as avoiding adverse bleeding, are primary concerns. We were encouraged that we measured ng/l quantities of diclofenac in serum from our rabbit model. The amounts measured are in keeping with published human studies [25]. In terms of preservation of residual renal function and adverse bleeding further reducing the topically applied dose would be welcomed and may with further work potentially allow such practice to be adopted clinically.

In the present study we have confirmed diclofenac mediated inhibition of VSM cell proliferation (Fig. 3A). The anti-proliferative mechanism of diclofenac is independent of the classic mitogenic signalling pathway ERK and p38 as diclofenac had no effect on phosphorylation of either component (Fig. 1B). Previously diclofenac has been shown to cause cell cycle arrest via activation of p53 leading to increased p21 [21]. The expression of cell cycle G1 associated cyclin D1 following diclofenac treatment was assessed in this study. Cyclin D1 is essential for cell cycle progression [27]. Diclofenac caused a concentration dependent decrease in cyclin D1 expression (Fig. 3C). The concentrations required for this activity (42.5–170 μM) were similar to those required for inhibition of VSM cell proliferation thus strongly indicating that diclofenac inhibits cell proliferation by reducing G1 associated cyclins, possibly via the p53–p21 pathway. The reported anti-proliferative IC_50_ of diclofenac required for anti-proliferative activity is 170 μM, which is vastly higher than the IC_50_ required for inhibition of COX-2 at 50 nM [21,28]. This conclusion supports the hypothesis that inhibition of cell proliferation by diclofenac is via a COX-independent means.

AMPK has previously been implicated in the NSAID aspirin's anti-proliferative activity [28]. In this study we have for the first time demonstrated that diclofenac mediates anti-proliferative activity through AMPK signalling. A diclofenac-dependent increase in AMPK phosphorylation was measured (Fig. 4A). To demonstrate progression of the AMPK pathway, phosphorylation of downstream protein ACC was also analysed. ACC is directly phosphorylated by AMPK to regulate the metabolism of fatty acids within the cell, but not thought to be involved in the regulation of cell cycle [29]. Diclofenac treatment resulted in a significant concentration dependent increase in ACC phosphorylation (Fig. 4A). The response generated was greater than that seen for AMPK activation, with an increase in phosphorylation occurring following 20 μM diclofenac treatment. Similar effects have been reported for aspirin and nifedipine, both of which activate AMPK causing amplified ACC activation [29-31].

To confirm the AMPK-dependent mechanism of action of diclofenac, a number of approaches were taken to block AMPK. Firstly, a pharmacological approach was taken using the AMPK antagonist compound C. This agent significantly inhibited diclofenac mediated AMPK activation, as well as downstream activation of ACC (Fig. 4C). The effect of compound C on diclofenac's anti-proliferative activity was evaluated. Initial experiments assessed the effect of compound C alone. In contrast to what was expected, compound C was shown to inhibit cell proliferation (Fig. 4D) suggesting that it may have a cytotoxic effect on the cells, or may have cellular substrates in addition to AMPK. However the viability of cells was not affected by compound C treatment, as demonstrated by trypan blue (Fig. 4E). Therefore the possibility that compound C may have non-AMPK specific effects was assessed. ERK, a component of a key signalling event in cell proliferation, was investigated. Compound C significantly inhibited FCS stimulated ERK phosphorylation (Fig. 4F). Importantly, this was in contrast to diclofenac treatment which had no effect on ERK (Fig. 4B). Thus compound C has AMPK independent effects and can reduce proliferation via inhibition of ERK.

Murine wild type and AMPKα1^−^/^−^ VSM cells where utilised to assess the impact of AMPK on diclofenac mediated inhibition of proliferation. This demonstrated for the first time that AMPKα1 was essential for diclofenac mediated activity. However, this study has not assessed the mechanism through which diclofenac activates AMPK. It is likely that the mechanism is similar to aspirin mediated allosteric activation of AMPK via binding of the beta subunit. As this is the first report of diclofenac mediated AMPK activation further studies are needed to establish this hypothesis.

## 5 Conclusions

This study has a number of novel observations; we have demonstrated that diclofenac causes a concentration dependent inhibition of VSM cell proliferation. The mechanisms of action are via an increase in AMPK phosphorylation and reduced expression of the cell cycle protein cyclin D1 and surprisingly, are independent of ERK/p38 mitogenic signalling. The mechanism of action underlying effects of diclofenac was confirmed in murine wild type and AMPKα1^−^/^−^ VSM cells where diclofenac mediated inhibition of proliferation was shown to be reliant on AMPKα1. However, the mechanism through which diclofenac activates AMPK is yet to be elucidated. The primary observation in this study was in our successful model of AVF creation in the adult rabbit. We have successfully demonstrated that cannulation results in cumulative injury that significantly contributes to adverse remodelling and luminal occlusion in the vein section of the surgically created AVFs. Importantly we have further demonstrated that prophylactic transdermal delivery of diclofenac to the AVF cannulation sight prevents cannulation-dependent remodelling. Further study is nonetheless required to define minimum effective dosage thus ensuring preservation of any residual kidney function in this vulnerable patient group. However this novel use of NSAIDs may offer a treatment option and if adopted could potentially prolong AVF patency.

## Conflict of Interest

The authors declare they have no conflict of interest.
